# Predictive factors for cerebrocardiac syndrome in patients with severe traumatic brain injury: a retrospective cohort study

**DOI:** 10.3389/fneur.2023.1192756

**Published:** 2023-07-19

**Authors:** Xin-Cai Wang, Shang-Jun Gao, Shi-Long Zhuo, Cui-Lian Weng, Hang-Wei Feng, Jian Lin, Xing-Sheng Lin, Long Huang

**Affiliations:** ^1^Department of Critical Care Medicine, Shengli Clinical Medical College of Fujian Medical University, Fujian Provincial Hospital South Branch, Fuzhou, China; ^2^Fujian Provincial Key Laboratory of Critical Care Medicine, Fuzhou, China; ^3^Department of Orthopedics, Shengli Clinical Medical College of Fujian Medical University, Fujian Provincial Hospital South Branch, Fuzhou, China; ^4^Department of School of Electronic, Electrical Engineering and Physics, Fujian University of Technology, Fuzhou, China

**Keywords:** cerebrocardiac syndrome, severe traumatic brain injury, optic nerve sheath diameter, Tei index, glasgow coma scale, cardiac troponin-I

## Abstract

**Background and objective:**

Cerebrocardiac syndrome (CCS) is a severe complication of severe traumatic brain injury (sTBI) that carries high mortality and disability rates. Early identification of CCS poses a significant clinical challenge. The main objective of this study was to investigate potential risk factors associated with the development of secondary CCS in patients with sTBI. It was hypothesized that elevated right heart Tei index (TI), lower Glasgow Coma Scale (GCS) scores, and elevated cardiac troponin-I (cTnI) levels would independently contribute to the occurrence of CCS in sTBI patients.

**Methods:**

A retrospective cohort study was conducted to identify risk factors for CCS secondary to sTBI. One hundred and fifty-five patients were enrolled with sTBI admitted to the hospital between January 2016 and December 2020 and divided them into a CCS group (*n* = 75) and a non-CCS group (*n* = 80) based on the presence of CCS. This study involved the analysis and comparison of clinical data from two patient groups, encompassing demographic characteristics, peripheral oxygen saturation (SPO2), neuron-specific enolase (NSE), cardiac troponin-I (cTnI), N-terminal pro-brain natriuretic peptide (NT-proBNP), optic nerve sheath diameter (ONSD), cardiac ultrasound, acute physiology and chronic health evaluation (APACHE II) scores, and GCS scores and so on. Multivariate logistic regression was employed to identify independent risk factors for CCS, and receiver operating characteristic (ROC) curves were used to assess their predictive value for CCS secondary to sTBI.

**Results:**

The study revealed that 48.4% of sTBI patients developed secondary CCS. In the multivariate analysis model 1 that does not include NT-proBNP and cTnI, ONSD (OR = 2.582, 95% CI: 1.054–6.327, *P* = 0.038), right heart Tei index (OR = 2.81, 95% CI: 1.288–6.129, *P* = 0.009), and GCS (OR = 0.212, 95% CI: 0.086–0.521, *P* = 0.001) were independent risk factors for secondary CCS in sTBI patients. In multivariate analysis model 2 that includes NT-proBNP and cTnI, cTnI (OR = 27.711, 95%CI: 3.086–248.795, *P* = 0.003), right heart Tei index (OR = 2.736, 95% CI: 1.056–7.091, *P* = 0.038), and GCS (OR = 0.147, 95% CI: 0.045–0.481, *P* = 0.002) were independent risk factors for secondary CCS in sTBI patients. The area under the ROC curve for ONSD, Tei index, GCS, and cTnI were 0.596, 0.613, 0.635, and 0.881, respectively. ONSD exhibited a positive predictive value (PPV) of 0.704 and a negative predictive value (NPV) of 0.634. The Tei index demonstrated a PPV of 0.624 and an NPV of 0.726, while GCS had a PPV of 0.644 and an NPV of 0.815. On the other hand, cTnI exhibited a significantly higher PPV of 0.936 and an NPV of 0.817. These findings indicate that the Tei index, GCS score, and cTnI possess certain predictive value for secondary CCS in patients with sTBI.

**Conclusions:**

The study provides valuable insights into the identification of independent risk factors for CCS secondary to sTBI. The findings highlight the significance of right heart Tei index, GCS score, and cTnI as potential predictive factors for CCS in sTBI patients. Further larger-scale studies are warranted to corroborate these findings and to provide robust evidence for the development of early intervention strategies aimed at reducing the incidence of CCS in this patient population.

## 1. Introduction

Traumatic brain injury (TBI) is a common and severe clinical condition with a high mortality rate. TBI severity is classified based on the Glasgow Coma Scale (GCS) scores, where mild TBI is defined as GCS 13–15, moderate TBI as GCS 9–12, and severe TBI as GCS 3–8. Cerebrocardiac syndrome (CCS) is an important complication of severe TBI. Understanding the relationship between TBI severity and the occurrence of CCS is crucial for improving clinical management and prognosis in TBI patients (1, 2). CCS is a collection of acute cardiac complications, including myocardial infarction, subendocardial hemorrhage, myocardial ischemia, arrhythmia, or heart failure, arising from acute encephalopathy (3, 4). Despite the high incidence of CCS secondary to sTBI, which occurs in 68–90% of cases (5), the underlying mechanisms remain poorly understood. Neurohumoral dysregulation, imbalances in brain regulation of cardiac activity, and cytokine release are thought to contribute to its development. sTBI, a common cause of CCS, is typically associated with car accidents and falls from heights (6, 7), and has a high mortality rate despite prompt surgical intervention and the use of hyperosmotic agents to decrease intracranial pressure (8). Notably, patients with sTBI who develop secondary CCS have an even higher mortality rate compared to those who do not (4). Identifying predictive risk factors has important clinical implications for early diagnosis and intervention, given the significant morbidity and mortality associated with CCS secondary to sTBI.

Currently, the diagnosis of CCS relies on patient history, clinical features, electrocardiograph (ECG) features, and markers of myocardial injury (3). The diagnosis of CCS should first exclude pre-existing organic lesions of the heart valves and myocardium prior to the onset of cerebrovascular disease, and at the same time, exclude history of arrhythmias. When CCS occurs in acute cerebrovascular disease, it is accompanied by symptoms of myocardial ischemia, myocardial infarction, and/or arrhythmias and corresponding ECG changes. With the exception of deceased patients, the acute cerebrovascular symptoms will gradually improve with CCS and eventually return to normality or minor residual abnormality. However, cerebrovascular and cardiovascular diseases often share a common pathological basis, such as hypertension and atherosclerosis, and comorbidity can mask each other or be mutually causal, affecting timely diagnosis and treatment. Additionally, some patients may be neglected because the symptoms and signs of early acute cerebrovascular disease are mild or nonexistent. Moreover, when chest tightness and palpitations are the main complaints, with ECG showing myocardial ischemia or injury and arrhythmia, clinicians may only consider coronary heart disease, making it difficult to recognize the existence of CCS caused by acute cerebrovascular disease, leading to misdiagnosis.

Little research has been focused on CCS. Dai et al. (9) analyzed the relationship between serum macrophage migration inhibitory factor (MIF) concentrations and CCS by recruiting groups of patients with sTBI and healthy subjects. This study found that patient concentrations were significantly higher than controls and that serum MIF concentrations were highly correlated with CCS, suggesting that elevated serum MIF may be a valid biomarker for early detection of CCS after traumatic brain injury. Based on the findings of this study, it is believed that further exploration of molecular markers with regulatory functions in CCS or clinical indicators with early predictive value may offer new insights for investigating early clinical treatment strategies for CCS.

In recent years, critical care ultrasound has gained increasing recognition among clinicians for its non-invasive, rapid, accurate, and dynamic continuous monitoring advantages, bringing significant convenience and greatly improving clinical diagnosis and treatment. Ultrasound has made significant advancements in the assessment of lung effusion (10), hemodynamic monitoring (11), critical care cranial ultrasound (12), and more. The brain and heart are the main organs involved in CCS, and critical care ultrasound has clear advantages in the assessment of intracranial pressure and cardiac systolic function (13). As the diagnosis of CCS lacks clear diagnostic indicators, relying only on patient history, clinical features, and ECG characteristics can lead to misdiagnosis and omission, hampering early diagnosis and timely treatment. Therefore, identifying early, noninvasive CCS warning indicators has become an urgent clinical objective. This study aims to collect clinical data from patients with sTBI, analyze independent risk factors for secondary CCS in sTBI, and investigate the value of noninvasive monitoring indices in assessing the factors contributing to secondary CCS in sTBI. Based on clinical observations, the hypothesis is that increased optic nerve sheath diameter (ONSD), elevated Tei index, and lower Glasgow Coma Scale (GCS) scores would serve as independent risk factors for the occurrence of CCS in patients with sTBI.

## 2. Materials and methods

### 2.1. Setting

This retrospective cohort study was conducted in two tertiary hospitals, namely the Department of Critical Care Medicine at Shengli Clinical Medical College of Fujian Medical University Fujian Provincial Hospital and Fujian Provincial Hospital South Branch in Fuzhou, China, spanning from January 2016 to December 2020. The study was approved by the Institution Review Board (IRB) of Shengli Clinical Medical College of Fujian Medical University under the ethical approval number K2021-04-079. Clinical data was collected solely from patients, without any interference with their treatment plans, thus posing no physiological risks to them. The IRB of Shengli Clinical Medical College of Fujian Medical University has granted an exemption of informed consent for this retrospective study. Moreover, all clinical data of patients was collected in accordance with the Declaration of Helsinki and the International Conference on Harmonization Guidelines for Good Clinical Practice.

### 2.2. Identification of the study population

All consecutive adult patients diagnosed with TBI were screened for the study. Patients eligible for this study had to meet all the following criteria (14): (1) age: 18–75 years old; (2) sTBI occurred to the time of hospital admission ≤ 6 h; (3) no serious trauma to other sites except craniocerebral trauma. Exclusion criteria include: (1) previous combined coronary artery disease and/or other heart valves abnormality, combined organic lesions of the heart; (2) combined malignancy and other serious underlying diseases of the liver, kidney and brain; (3) a diagnosis of mild TBI or moderate TBI. The diagnostic criteria for CCS used in this study are (15, 16): (1) the presence of non-low potassium U waves, ST-segment depression, and atrioventricular block on the electrocardiogram; (2) abnormal levels of biomarkers of cardiac injury, such as N-terminal pro-brain natriuretic peptide (NT-proBNP) (>125 pg/ml) and/or cardiac troponin-I(cTnI) (>0.04 ng/ml).

### 2.3. Data collection

The electronic medical records of patients diagnosed with severe traumatic brain injury (sTBI) were meticulously examined to collect data on several variables, including age, gender, smoking and alcohol consumption, and underlying conditions such as hypertension and/or diabetes. Furthermore, data recorded at admission, including peripheral oxygen saturation (SPO_2_), heart rate, use of vasoactive medications, cranial CT imaging, GCS scores, and Acute Physiology and Chronic Health Evaluation (APACHE II) scores were collected, along with post-admission blood tests, bedside cardiac ultrasound parameters, intracranial pressure (ICP), ONSD at corresponding times after cranial surgery, and other pertinent clinical information.

**Figure 1:**
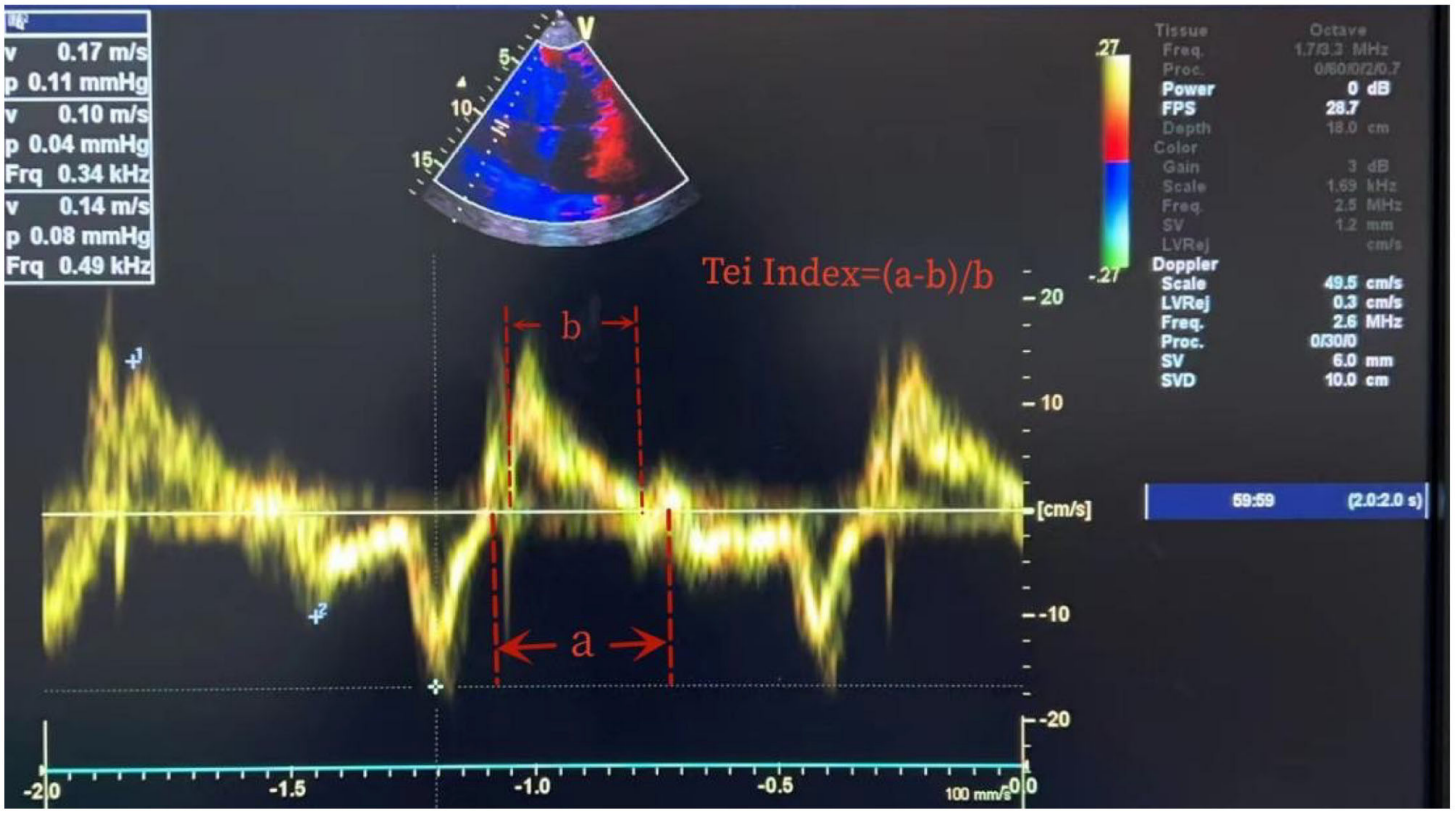
Measurement of Tei index. Tei index = (ICT + RCT)/ET. ICT, isovolumic contraction time; RCT, isovolumic relaxation time; ET, ejection time. As ICT + RCT = a-b and ET = b, the Tei index is (a-b)/b.

**Figure 2:**
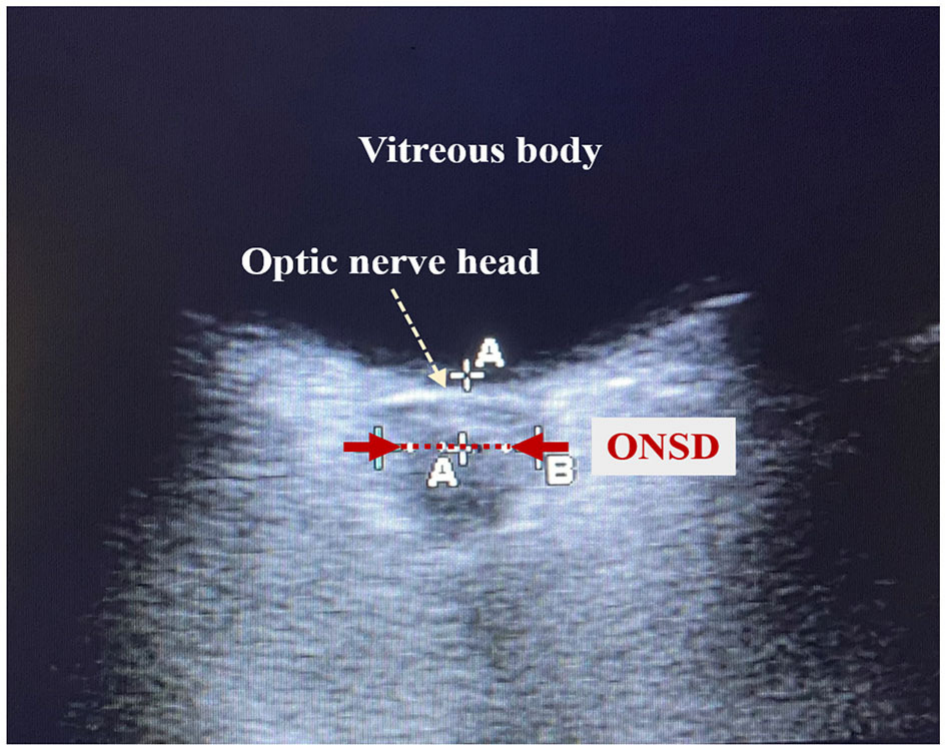
Schematic diagram of optic nerve sheath diameter measurement. The red dotted line shown by the red arrow is the optic nerve sheath. The length of the red dotted line is the optic nerve sheath diameter (ONSD).

The clinicians who conducted the cardiac ultrasound and ONSD measurements were trained and certified in critical care ultrasound within the department. Cardiac ultrasound measurements were conducted using an EDGE-type color Doppler ultrasound machine (Sono Sound) to obtain parameters such as isovolumic contraction time (ICT), isovolumic relaxation time (IRT), ejection time, and the Tei index, which is a time interval index derived from Doppler ultrasound, calculated as the sum of ICT and IRT divided by ET (Figure 1). The ONSD measurements were conducted using a high-frequency (5.0–10.5 MHz) line array ultrasound probe from an EDGE-type color Doppler ultrasound machine (Sono Sound) to measure the width of the optic nerve sheath at the posterior 3 mm of the eye. The width of the sheath was measured, and the average value was obtained by repeating the measurement twice (Figure 2).

### 2.4. Statistical processing

Statistical analysis for this study was performed using SPSS 25.0 software. Non-normally distributed variables were reported as M (P25, P75), and the Mann-Whitney U test was used for between-group comparisons. Count data were presented as the number of cases (%), and the chi-square test was used for group comparisons. Spearman correlation analysis was employed to investigate the linear relationship between non-normally distributed continuous variables. Univariate analysis was conducted to compare clinical data of patients in the CCS and non-CCS groups, and variables exhibiting significant differences were entered into a dichotomous logistic regression model to analyze the independent risk factors for sTBI secondary to CCS. Predictive value of the parameters on the variables for CCS was determined using the receiver operating characteristic curve (ROC). A *P* < 0.05 was considered statistically significant.

## 3. Results

### 3.1. Comparison of sTBI patient general information

**Figure 3:**
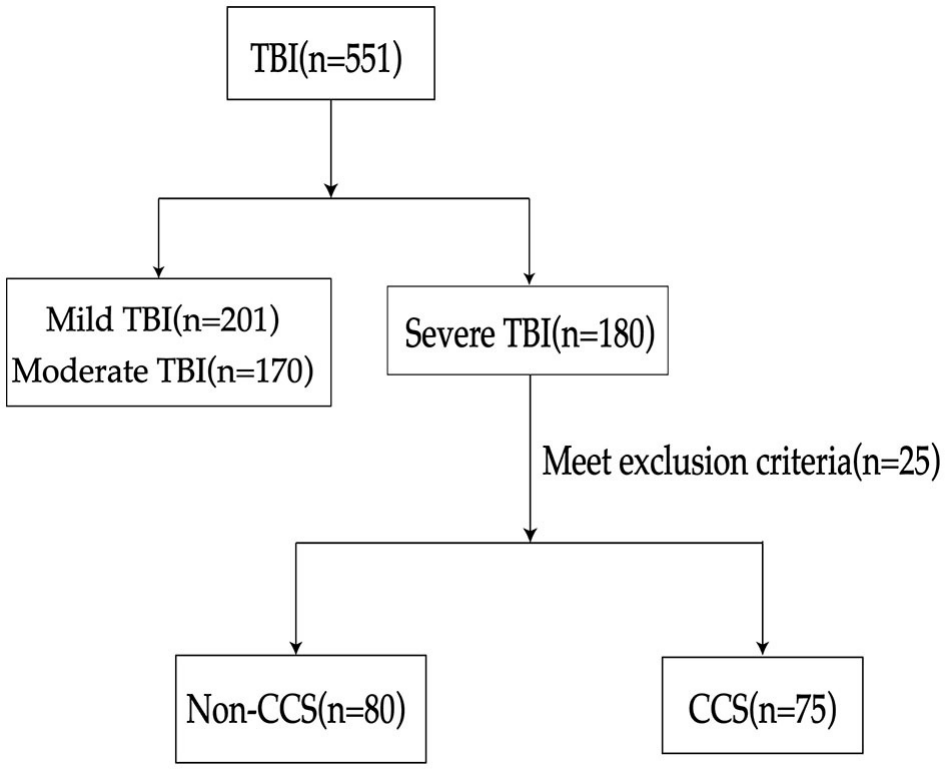
Flowchart of patient selection process. TBI, traumatic brain injury; CCS, cerebrocardiac syndrome.

In this retrospective cohort study, ~551 patients diagnosed with TBI were screened, among which 155 were confirmed cases of sTBI (Figure 3). These patients were subsequently divided into two groups, namely the CCS group and the non-CCS group, based on whether they had secondary CCS. Among them, the number of patients with sTBI secondary to CCS was 75, accounting for 48.4% of the total number of patients with sTBI (75/155). The results indicate that the APACHE II score, ICP, ONSD, right heart Tei index, and NSE levels were significantly higher in the CCS group compared to the non-CCS group (*P* < 0.05; Table 1), while the GCS of patients in the CCS group was significantly lower than that of patients in the non-CCS group (*P* < 0.01; Table 1).

**Table 1:** Comparison of clinical indicators between the non-CCS and CCS groups in patients with sTBI [*n* (%)/M (P 25, P 75)].

**Clinical indicators**	**sTBI patients**		***P-*value**
	**Non-CCS group** ***n*** = **80**	**CCS group** ***n*** = **75**	
**Gender**			
Male	52	44	0.417^a^
Female	28	31	
Age	54 (39.25, 59.75)	53 (39, 65)	0.56
Smoking	30 (37.5)	26 (34.7)	0.714
Drinking alcohol	25 (31.2)	16 (21.3)	0.162
SpO2, %	96.0 (93.0, 97.2)	96.0 (93.0, 98.0)	0.808
Heart rate, bpm	91.0 (80.8, 110.0)	86.0 (78.0, 107.5)	0.417
Vasoactive medications	14 (17.5)	11 (14.7)	0.417
**Positive imaging findings**			
Epidural hematoma	48 (60.0)	49 (65.3)	0.603
Subdural hematoma	34 (42.5)	39 (52.0)	0.306
Basal fracture	38 (47.5)	42 (56.0)	0.369
Calvarial fractures	51 (63.8)	41 (54.7)	0.324
Cerebral contusion	55 (68.8)	54 (72.0)	0.790
Subarachnoid hemorrhage	59 (73.8)	52 (69.3)	0.666
Midline shift >5 mm	54 (67.5)	51 (68.0)	1.000
Hypertension	15 (19.0)	15 (20.0)	0.874
Diabetes	6 (7.5)	7 (9.5)	0.662
APACHE II	31 (28, 34)	32 (31, 34)	0.044
NSE, ng/mL	18 (15.19, 26.5)	45 (20.2, 116)	0.000
LDH, IU/L	223 (191, 288)	267 (206.5, 345.5)	0.062
CK-MB, IU/L	23 (16, 35.5)	25 (17.5, 53)	0.171
ICP, mmHg	16 (11, 18)	19 (12, 23)	0.013
ONSD, mm	4.6 (4.5, 4.8)	4.9 (4.3, 5.1)	0.039
EF, %	53 (52, 55.75)	53 (52, 55)	0.187
VTI	23 (22, 24)	23 (22, 24)	0.796
MAPSE	16 (14, 16)	16 (14, 17)	0.896
E/A	1.3 (1.2, 1.5)	1.4 (1.3, 1.5)	0.172
E/e'	12 (9.5, 13)	12 (10, 14)	0.317
Tei Index	0.41 (0.37, 0.42)	0.43 (0.40, 0.44)	0.015
TAPSE	18 (14.25, 21.00)	16 (14, 21)	0.357
GCS Score	9 (6, 10)	7 (6, 8)	0.004
NT-proBNP	127 (78.0,239.5)	822 (195.4,1920.0)	< 0.001
cTnI	0.0 (0.0,0.1)	0.1 (0.0,0.3)	< 0.001

^a^Chi square analysis was employed and the rest uses Mann–Whitney U-test.

SpO2, peripheral oxygen saturation; APACHE II, acute physiology and chronic health evaluation; NSE, neuron specific enolase; LDH, lactate dehydrogenase; CK-MB, creatine kinase-MB; ICP, intracranial pressure; ONSD, optic nerve sheath diameter, EF, ejection fraction; VTI, velocity time integral; MAPSE, mitral annular plane systolic excursion; E, peak early diastolic transmitral flow; A, peak late diastolic transmitral flow velocit; e', peak early diastolic mitral annulus velocity; TAPSE, tricuspid annular plane systolic excursion; GCS, glasgow coma scale; NT-proBNP, N-terminal pro-brain natriuretic peptide; cTnI, cardiac troponin-I.

**Figure 4:**
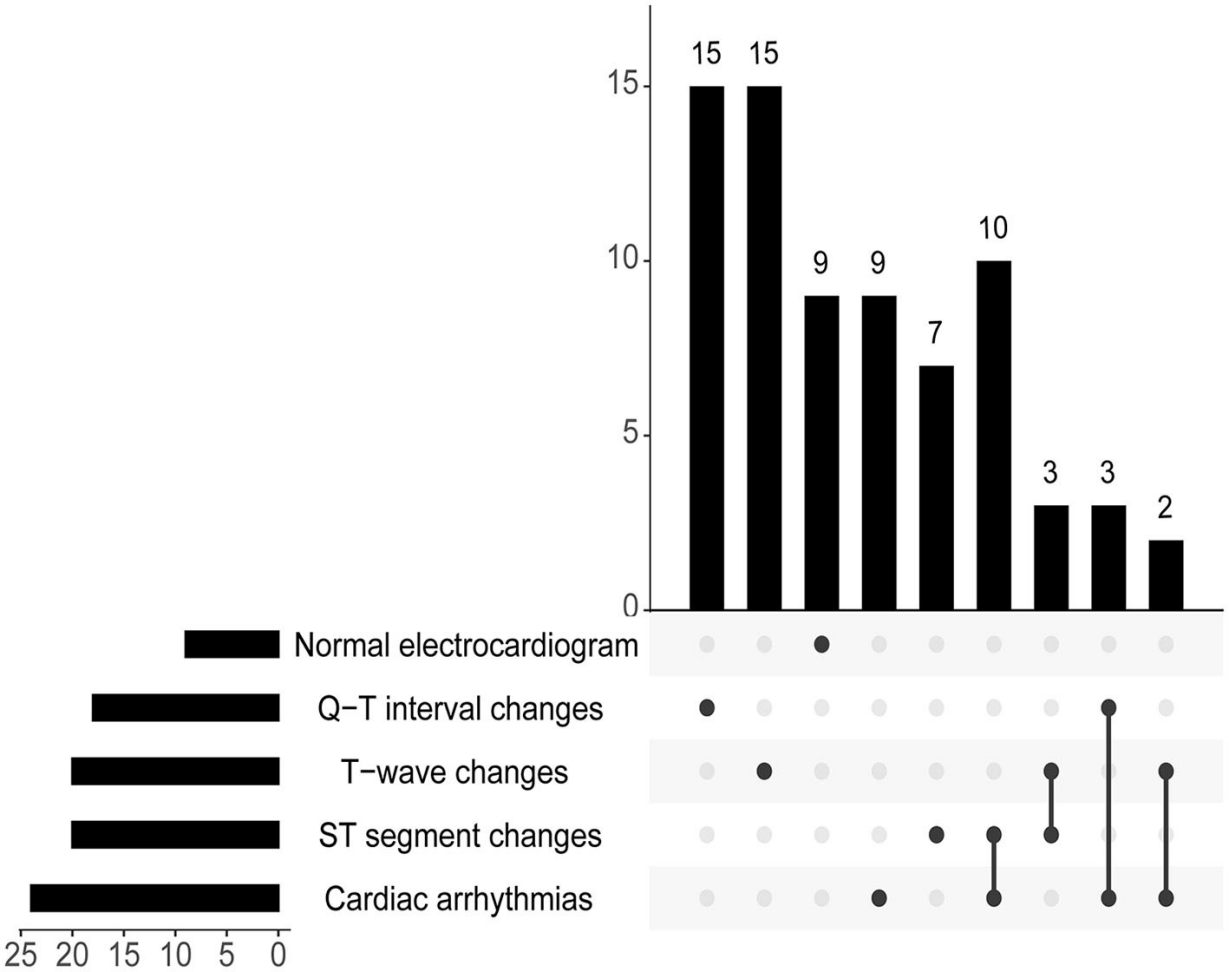
The electrocardiogram presentation of CCS is depicted as an upset pattern.

In the Non-CCS Group, there were five patients who developed sinus bradycardia due to intracranial hypertension. In the study, among patients who have already experienced CCS, nine patients had a normal electrocardiogram, 24 patients experienced cardiac arrhythmias, 20 patients exhibited T-wave changes, 20 patients showed ST segment changes, and 18 patients had Q-T interval changes (Figure 4).

**Figure 5:**
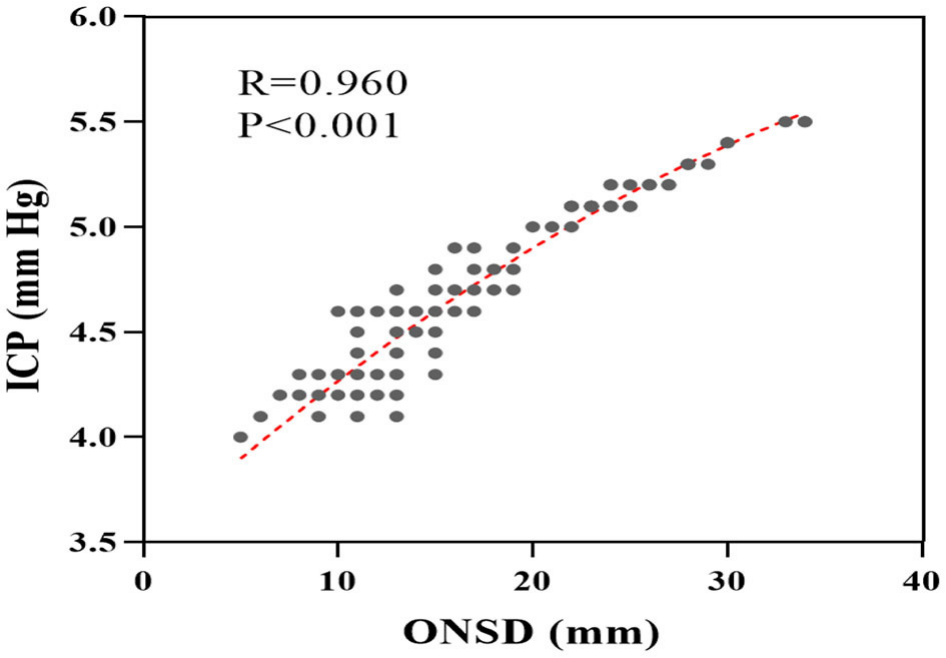
Scatter plot of correlation between ICP and ONSD. ONSD, optic nerve sheath diameter; ICP, intracranial pressure.

It is worth noting that several studies have demonstrated the superiority of ONSD in dynamically reflecting ICP levels (17–20). In the study, a scatter plot was generated using the collected data to illustrate the relationship between ONSD and ICP. As depicted in Figure 5, a positive correlation was observed between ONSD and ICP. The Spearman correlation analysis revealed a correlation coefficient of *R* = 0.960 (95% CI: 0.95–0.97, *P* < 0.001), which indicates a significant association between ICP and ONSD.

### 3.2. Identification of risk factors for sTBI secondary to CCS

To further elucidate the independent risk factors for sTBI secondary to CCS, a multivariate logistic regression analysis was conducted, including all potential confounding variables with a *P* < 0.05 in the univariate analysis (Table 1). Due to NT-proBNP and cTnI being regarded as essential diagnostic markers for CCS in this study, we opted not to include them in the multivariate analysis of Model 1 (Table 2). Both ICP and ONSD serve as indicators of intracranial pressure. Given their significant correlation, the subsequent multifactorial logistic analysis focused solely on the noninvasive monitoring indicator ONSD. This approach aimed to assess the value of noninvasive monitoring indicators in the evaluation of secondary CCS in sTBI, without considering the invasive measure of ICP. The results in Table 2 demonstrate that optic nerve sheath diameter (ONSD, OR = 2.582, 95% CI: 1.054–6.327, *P* = 0.038), right heart Tei index (OR = 2.81, 95% CI: 1.288–6.129, *P* = 0.009), and GCS (OR = 0.212, 95% CI: 0.086–0.521, *P* = 0.001) remained significantly correlated with the occurrence of CCS. Therefore, in Model 1, ONSD, right heart Tei index, and GCS were identified as independent risk factors for CCS secondary to sTBI.

**Table 2:** Model 1: multivariable logistic regression analysis of sTBI secondary to CCS.

**Variable**	***OR***	**95% *CI***	***P***
NSE	2.963	0.725–12.112	0.130
ONSD	2.582	1.054–6.327	0.038
Tei index	2.810	1.288–6.129	0.009
APACHE II	0.940	0.813–1.088	0.408
GCS	0.212	0.086–0.521	0.001

NSE, neuron specific enolase; ONSD, optic nerve sheath diameter; APACHE II, acute physiology and chronic health evaluation; GCS, glasgow coma scale.

In order to comprehensively account for potential predictors of CCS and enhance the robustness of our findings, Model 2 of this study incorporated all potential confounding variables with a statistically significant *P* < 0.05 from the univariate analysis (Table 3). This approach aimed to capture a wide range of influential factors and minimize the potential for omitted variable bias, thereby strengthening the validity and reliability of our results. The results presented in Table 3 indicate a significant correlation between the occurrence of CCS and cTnI (OR = 27.711, 95%CI: 3.086–248.795, *P* = 0.003), right heart Tei index (OR = 2.736, 95% CI: 1.056–7.091, *P* = 0.038), and GCS (OR = 0.147, 95% CI: 0.045–0.481, *P* = 0.002). These findings suggest that cTnI, right heart Tei index, and GCS are independent risk factors for CCS secondary to sTBI, as determined by Model 2 analysis.

**Table 3:** Model 2: multivariable logistic regression analysis of sTBI secondary to CCS.

**Variable**	***OR***	**95% *CI***	***P***
NSE	2.014	0.412–9.833	0.387
ONSD	2.417	0.831–7.026	0.105
Tei index	2.736	1.056–7.091	0.038
APACHE II	0.834	0.691–1.013	0.051
GCS	0.147	0.045–0.481	0.002
cTnI	27.711	3.086–248.795	0.003
NT-proBNP	1.003	0.998–1.002	0.733

NSE, neuron specific enolase; ONSD, optic nerve sheath diameter; APACHE II, acute physiology and chronic health evaluation; GCS, glasgow coma scale; cTnI, cardiac troponin-I; NT-proBNP, N-terminal pro-brain natriuretic peptide.

### 3.3. Predictive value of independent risk factors for secondary CCS in patients with sTBI

**Figure 6:**
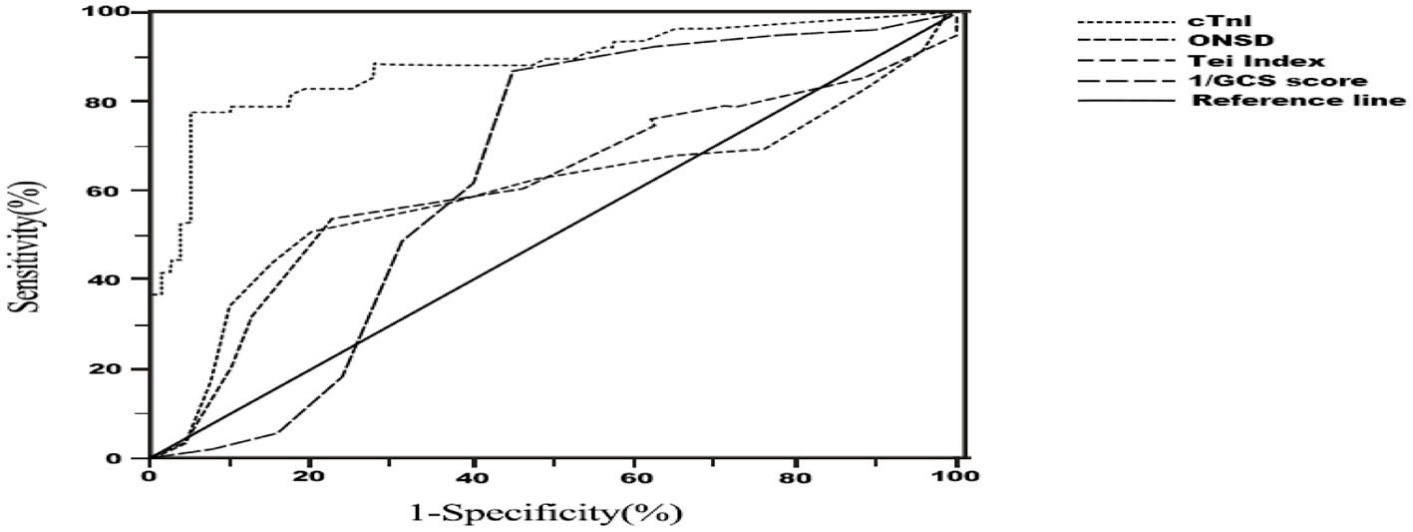
Receiver Operator Characteristic (ROC) curve for diagnosis of cerebrocardiac syndrome secondary to sever traumatic brain injury. ONSD, optic nerve sheath diameter; GCS, glasgow coma scale; cTnI, cardiac troponin-I.

In Table 4, the AUC values of each independent risk factor for predicting sTBI secondary CCS were determined. The AUC value for ONSD was 0.596 (95% CI: 0.502–0.690, *P* = 0.039), with a specificity of 80.0% and sensitivity of 50.7% when ONSD ≥ 4.85 mm. The AUC value for right heart Tei index was 0.613 (95% CI: 0.521–0.704, *P* = 0.016), with a specificity of 53.3% and sensitivity of 77.5%. The AUC value for GCS was 0.635 (95% CI: 0.543–0.727, *P* = 0.004), with a specificity of 55.0% and sensitivity of 86.7% when GCS was ≤ 8.47. The AUC value for cTnI was 0.881 (95% CI: 0.825–0.937, *P* = 0.003), with a specificity of 78.7% and sensitivity of 88.0% when GCS was ≥0.04 ng/ml. The positive predictive value (PPV) of ONSD is 0.704, and the negative predictive value (NPV) is 0.634. The PPV of the Tei index is 0.624, and the NPV is 0.726. The PPV of GCS is 0.644, and the NPV is 0.815. The PPV of cTnI is 0.936, and the NPV is 0.817. These results suggest that ONSD, right heart Tei index, GCS and cTnI all have good predictive value for assessing and predicting CCS secondary to sTBI, as shown in Figure 6.

**Table 4:** ONSD, Tei index, GCS, and cTnI to predict the efficacy of sTBI secondary to CCS.

**Indicator**	***AUC***	**95%CI**	**Cut-off point**	**Specificity (%)**	**Sensitivity (%)**
ONSD	0.596	0.502–0.690	4.850	50.7	80.0
tei index	0.613	0.521–0.704	0.425	53.3	77.5
GCS	0.635	0.543–0.727	8.47	55.0	86.7
cTnI	0.881	0.825–0.937	0.04	78.7	88.0

ONSD, optic nerve sheath diameter; GCS, glasgow coma scale; cTnI, cardiac troponin-I; AUC, area under the curve.

## 4. Discussion

sTBI is a common clinical condition associated with high mortality and disability rates (4). CCS is a frequent complication of sTBI and results in over 1.5 million deaths globally each year (5). Despite advances in medical knowledge and technology, the diagnosis and treatment of CCS still present significant challenges in clinical practice. Early predictive assessment of CCS occurrence and timely treatment can effectively improve patient prognosis. The study found that the incidence of CCS in 155 patients with sTBI was 48.4%, which is consistent with previously reported rates (21). However, it has been mentioned in the literature that moderate to sTBI can affect left ventricular ejection fraction, with ~22% of patients experiencing abnormal left ventricular systolic function. It suggests a direct correlation between the severity of brain injury and cardiac dysfunction (22). However, these studies did not explicitly state whether these patients met the definition of CCS. The study focused on including patients with sTBI, a population known for higher incidence and mortality rates of CCS. This finding further confirms the direct impact of brain injury severity on cardiac function.

The optic nerve sheath is a multi-layered structure composed of three meningeal layers. Durouchoux et al. (23) have previously shown that the ONSD in sTBI patients positively correlates with intracranial pressure, suggesting that ONSD may serve as an indicator of the severity of cranial injury to some extent. Various studies, including those on animal models, *ex vivo* optic nerve sheath experiments, and fresh autopsy ONSD, have shown a positive correlation between ONSD and intracranial pressure (21, 24, 25). A meta-analysis by Robba et al. (18) suggests that ONSD monitoring is non-invasive, simple, and easy to perform at bedside, and reduces the need for invasive procedures and the risk of infection. The present study further confirms the significant correlation between ICP and ONSD (*R* = 0.960), which is consistent with previous findings. Elevated ONSD indicates an increase in intracranial pressure, which can lead to cerebral ischemia and hypoxia, and ultimately to CCS. However, Hansen et al. (26) conducted *in vitro* experiments and discovered that when ICP exceeds 45 mmHg, the ONSD rapidly expands. This can reduce the correlation between ICP and ONSD because of the expandable space and reversibility of ONSD. Consequently, in cases of sudden ICP elevation, the clinical significance of ONSD as an indicator of intracranial pressure levels should be evaluated in combination with the patient's clinical symptoms and imaging to make a comprehensive judgment.

Multivariate logistic regression model 1 analysis in this study further indicates that ONSD is an independent risk factor for predicting the occurrence of CCS, and that ONSD ≥ 4.85 mm has a high specificity of 80%. Although there is currently no unified standard for the normal and critical values of ONSD measured by ultrasound, the findings of this study are expected to provide some clinical basis for establishing critical values for ONSD.

However, the AUC for ONSD in this study was not high. When ONSD was included in multivariate analysis using Model 2, which included cTnI and NT-proBNP, the results suggested that ONSD was not a stable independent risk factor for predicting CCS. This finding may be partly attributed to the relatively small sample size of this study. Nevertheless, the strong correlation between ONSD and ICP indicates that it still holds clinical value as a non-invasive tool for predicting CCS.

In the model 2, this study uncovered cTnI as an autonomous risk factor for CCS onset, demonstrating its remarkable predictive potency. This finding further substantiates previous research, such as the studies conducted by El-Menyar and Sezer, which demonstrated elevated expression of high-sensitivity troponin T (HsTnT) and cTnI in patients with TBI. These biomarkers hold potential as valuable tools for early risk stratification and prompt intervention in TBI (27, 28).

On the other hand, the right heart Tei index is an important indicator for monitoring cardiac function, providing a comprehensive reflection of cardiac diastolic function without interference from age and heart rate, and is highly operable (29, 30). Previous studies have shown that the right heart Tei index can be used to assess the severity and prognosis of septic cardiomyopathy (31), as well as pulmonary artery pressure in patients with connective tissue disease secondary to pulmonary hypertension (32). Despite the rarity of specific cardiac ultrasound variables as independent predictors of cardiac dysfunction after TBI (33), the predictive ability of the Tei index in CCS secondary to TBI has not been extensively studied. This study demonstrates that the right heart Tei index is an independent risk factor for predicting CCS with high sensitivity and specificity. Due to neurohumoral imbalances resulting from trauma, rehydration, and high doses of hyperosmotic treatment, the right heart, due to its anatomical characteristics, may not tolerate rapidly changing fluid imbalances and is more likely to display abnormalities earlier than the left ventricular EF and biomarkers of cardiac injury, potentially allowing for early prediction of sTBI secondary to a CCS event.

This study revealed that both the right heart Tei index and GCS serve as independent risk factors for predicting CCS, demonstrating consistent performance in multivariable analysis models 1 and 2. Moreover, these non-invasive measures, namely Tei index and GCS, exhibit promising potential for clinical application in the prediction of CCS.

The GCS was introduced in 1974 by two neurosurgery professors at the University of Glasgow and is currently the most widely used standardized method for assessing the condition of brain injury (34). Lower GCS scores indicate more severe coma, and some researchers have suggested that GCS scores can predict the prognosis of patients with sTBI (35). In the study, it was observed that GCS scores were lower in the CCS group compared to the non-CCS group, indicating an association between lower GCS scores and a higher risk of CCS. This finding further highlights the close relationship between the severity of craniocerebral injury and the occurrence of CCS.

NSE is a protein that is present in nerve cells and neuroendocrine cells, and is released into the blood when neurons are damaged (36). Gao et al. (37) demonstrated that NSE levels were significantly higher in patients with severe traumatic brain injury compared to those with common craniocerebral injury, and high expression of NSE was positively correlated with poor prognosis. Eric et al. (38) showed that NSE levels decreased gradually over time in patients with sTBI. In the study, NSE levels and APACHE II scores showed significant differences in univariate analysis, but did not demonstrate statistical significance when included in the multivariate regression model. This may be due to variations in the duration of onset in many patients, resulting in inconsistent changes in NSE levels, and the lack of dynamic and continuous monitoring of NSE and APACHE II scores.

However, it is important to acknowledge the limitations of this study, including its retrospective design, relatively small sample size, and insufficient study power, which may introduce potential selection bias. It is worth noting that the AUC values, as well as NPV and PPV values, of ONSD, right heart Tei index, and GCS in predicting CCS are relatively low, which may affect the stability of the conclusions. Furthermore, due to the retrospective nature of the study, the dynamic changes of ONSD over time could not be captured. Consequently, the exploration of progressive alterations of ONSD in CCS disease was limited. Therefore, further validation of these results is warranted through larger multicenter prospective studies.

## 5. Conclusion

The study provides valuable insights into the identification of independent risk factors for CCS secondary to sTBI. The findings highlight the significance of right heart Tei index, GCS score, and cTnI as potential predictive factors for CCS in sTBI patients.

## Data availability statement

The original contributions presented in the study are included in the article/supplementary material, further inquiries can be directed to the corresponding author.

## Ethics statement

The studies involving human participants were reviewed and approved by the IRB of Shengli Clinical Medical College of Fujian Medical University with the ethical approval number K2021-04-079. Written informed consent for participation was not required for this study in accordance with the national legislation and the institutional requirements.

## Author contributions

LH contributed to conception and design of the study. S-LZ and C-LW organized the database. H-WF, JL, and X-SL performed the statistical analysis. X-CW and S-JG wrote the first draft of the manuscript. All authors contributed to manuscript revision, read, and approved the submitted version.
